# Epidemiological and genomic characteristics of *Acinetobacter baumannii* from different infection sites using comparative genomics

**DOI:** 10.1186/s12864-021-07842-5

**Published:** 2021-07-12

**Authors:** Xingchen Bian, Xiaofen Liu, Xuefei Zhang, Xin Li, Jing Zhang, Huajun Zheng, Sichao Song, Xiang Li, Meiqing Feng

**Affiliations:** 1 School of Pharmacy & Minhang Hospital, Fudan University, 826 Zhang Heng Rd, 201203 Shanghai, China; 2 Institute of Antibiotics, Huashan Hospital, Fudan University, 200040 Shanghai, China; 3 Key Laboratory of Clinical Pharmacology of Antibiotics, 200040 Shanghai, China; 4 Huashan Hospital, National Health Commission & National Clinical Research Center for Aging and Medicine, Fudan University, 200040 Shanghai, China; 5 Phase I Unit, Huashan Hospital, Fudan University, 200040 Shanghai, China; 6 Chinese National Human Genome Center, 201203 Shanghai, China

**Keywords:** *Acinetobacter baumannii*, Whole genome sequencing, Epidemiological characteristics, Multi-drug resistance, Comparative genomics

## Abstract

**Background:**

*Acinetobacter baumannii* is a common nosocomial pathogen that poses a huge threat to global health. Owing to the severity of *A. baumannii* infections, it became necessary to investigate the epidemiological characteristics of *A. baumannii* in Chinese hospitals and find the reasons for the high antibiotic resistance rate and mortality. This study aimed to investigate the epidemiologic and genetic characteristics of *A. baumannii* isolated from patients with hospital acquired pneumonia (HAP), bloodstream infection (BSI) and urinary tract infection (UTI) in China and uncover potential mechanisms for multi-drug resistance and virulence characteristics of *A. baumannii* isolates.

**Results:**

All isolates were classified into two primary clades in core gene-based phylogenetic relationship. Clonal complex 208 (CC208) mainly consisted of ST195 (32 %) and ST208 (24.6 %). CC208 and non-CC208 isolates had carbapenem resistance rates of 96.2 and 9.1 %, respectively. Core genes were enriched in ‘Amino acid transport and metabolism’, ‘Translation’, ‘Energy production and conversion’, ‘Transcription’, ‘Inorganic ion transport and metabolism’ and ‘Cell wall/membrane/envelope synthesis’. Most isolates possessed virulence factors related to polysaccharide biosynthesis, capsular polysaccharide synthesis and motility. Eleven isolates belong to ST369 or ST191 (oxford scheme) all had the virulence factor *cap8E* and it had a higher positive rate in UTI (35.3 %) than in BSI (18.9 %) and HAP (12.9 %). ABGRI1 antibiotic resistance islands were responsible for streptomycin, tetracycline and sulfonate resistance. The *bla*_OXA−23_ gene was the most probable cause for carbapenem resistance, although the *bla*_OXA−66_ gene with nonsynonymous SNPs (F82L, I129L) was not.

**Conclusions:**

*A. baumannii* is a genomically variable pathogen that has the potential to cause a range of infectious diseases. There is high proportion of carbapenem resistance in isolates from all three infection sites (HAP, BSI and UTI), which can be attributed to the *bla*_OXA−23_ gene. CC208 is the predominant clone in *bla*_*OXA−23*_-carrying *A. baumannii* that should be monitored. Virulence factors involving bacteria motility and polysaccharide biosynthesis which are widespread in clinical *A. baumannii* strains deserve our attention.

**Supplementary Information:**

The online version contains supplementary material available at 10.1186/s12864-021-07842-5.

## Background

*Acinetobacter baumannii* has emerged as a dominant opportunistic Gram-negative bacterium causing a wide range of nosocomial infections [1]. The carbapenem resistance rate of *A. baumannii* reached nearly 75 % in response to treatment with imipenem or meropenem in China during 2019 (http://www.chinets.com/). Of particular concern is the mortality rate of ventilator associated pneumonia (VAP) in intensive care units (ICU), which ranges from 45.6 to 60.9 % and even reaches 84.3 % when VAP is caused by extensively drug-resistant *A. baumannii* (XDRAB) [2]. Owing to the severe consequences of *A. baumannii* infection, it is necessary to investigate its epidemiological characteristics and explore potential causes for the high antibiotic resistance rate and severe infections.

Several studies have focused on the molecular epidemiology of clinical *A. baumannii*. In northern China, ST191 and ST195 are the most common sequence types (STs) belonging to clonal complex 92 (also known as CC208). All of the *A. baumannii* isolates of these two STs contained carbapenem resistance gene *bla*_OXA−23_ [3], while ST208 and ST191 are likely the most common STs in southern China [4, 5].

Several studies investigated the molecular epidemiology of *A. baumannii*, focusing on its carbapenem resistance and virulence [6–8]. Few studies pay attention to the whole genome characteristics of clinical isolates from diverse infection sites. Investigating the phenotype and genotype characteristics of pathogens (i.e. phylogenetic relationship, sequence types, resistance and virulence genes) from different infections may provide meaningful information for empirical clinical medications, thus reducing the resistance transmission and mortality caused by hypervirulent strains. In our study, differential genetic characteristics were analysed among 64 isolates from hospital acquired pneumonia (HAP), bloodstream infection (BSI), or urinary tract infection (UTI). The epidemiological characteristics, resistance and virulence mechanisms were investigated according to draft genomes. Comparative genomic analysis was conducted in order to identify the structural and functional genomic relationship among these isolates.

## Results

### In vitro susceptibility of ***A. baumannii*** to antimicrobial agents

**Fig. 1:**
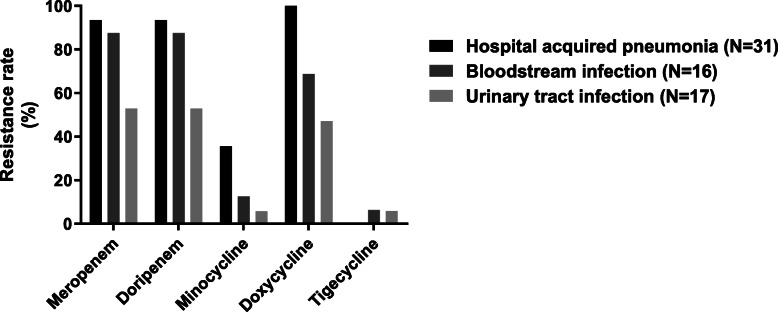
Resistance rates of *A. baumannii* to antimicrobials. No resistance was detected in colistin or polymyxin B. The resistance of tigecycline is determined according to FDA interpretation criteria for *Enterobacteriaceae*. There is no breakpoint for aztreonam and sulbactam

The resistance rates of clinical isolates of *A. baumannii* against antimicrobial agents are shown in Fig. 1. No polymyxins-resistant isolates were detected. Divided by infection types, isolates from HAP exhibited the highest resistance rates to other antimicrobial agents, while isolates from UTIs showed the lowest. The specific MIC values are presented in Table S4.

### Functional annotation of the genomic sequence of 64 ***A. baumanii*** isolates

**Fig. 2:**
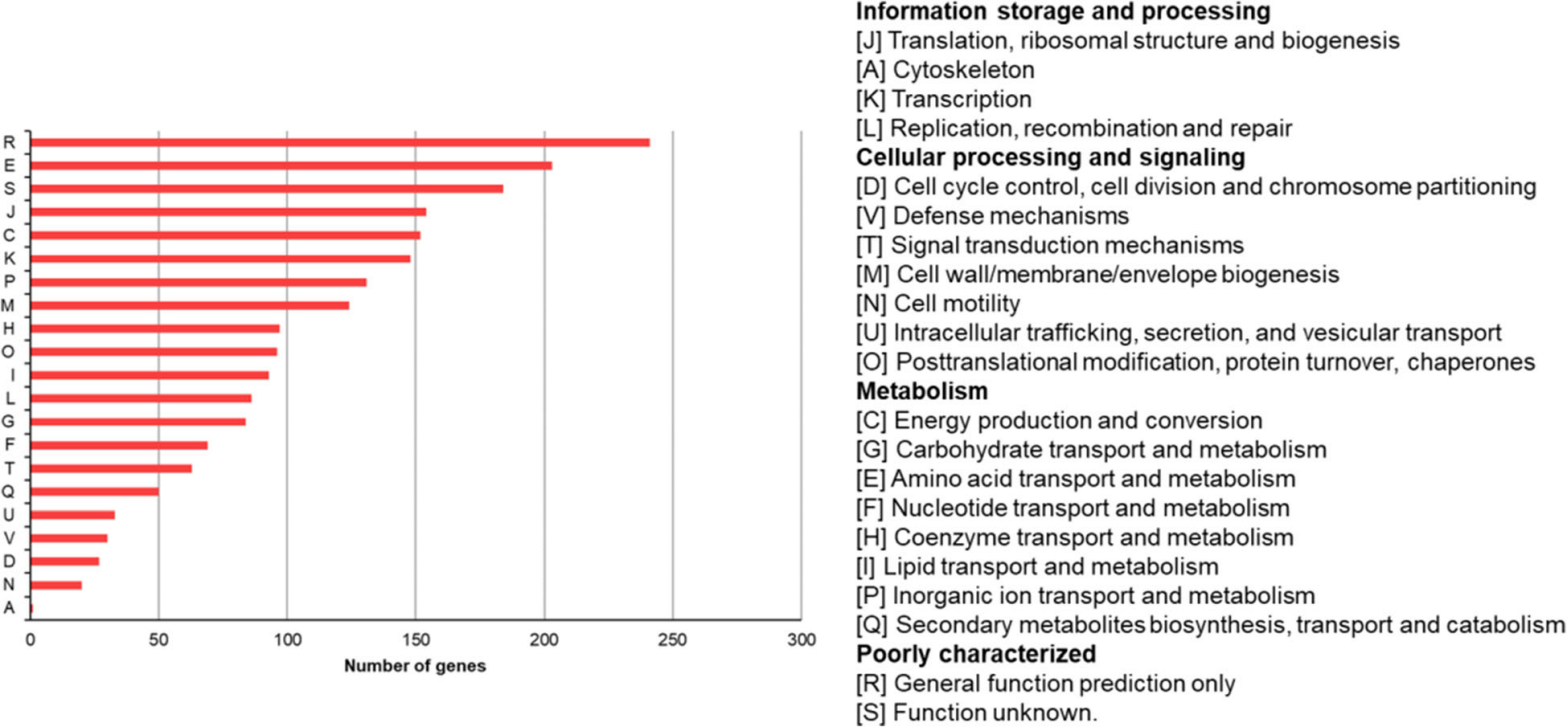
The distribution of COG categories of core genes of *A. baumannii*

Table S1 provides the quality information of sequencing data. Among 2403 core genes, 2086 (86.8 %) in total were annotated into 21 COG terms (Fig. 2). Most of them were annotated into ‘Amino acid transport and metabolism’, ‘Translation’, ‘Energy production and conversion’, ‘Transcription’, ‘Inorganic ion transport and metabolism’ and ‘Cell wall/membrane/envelope synthesis’.

### Virulence genes and infection sites

**Fig. 3:**
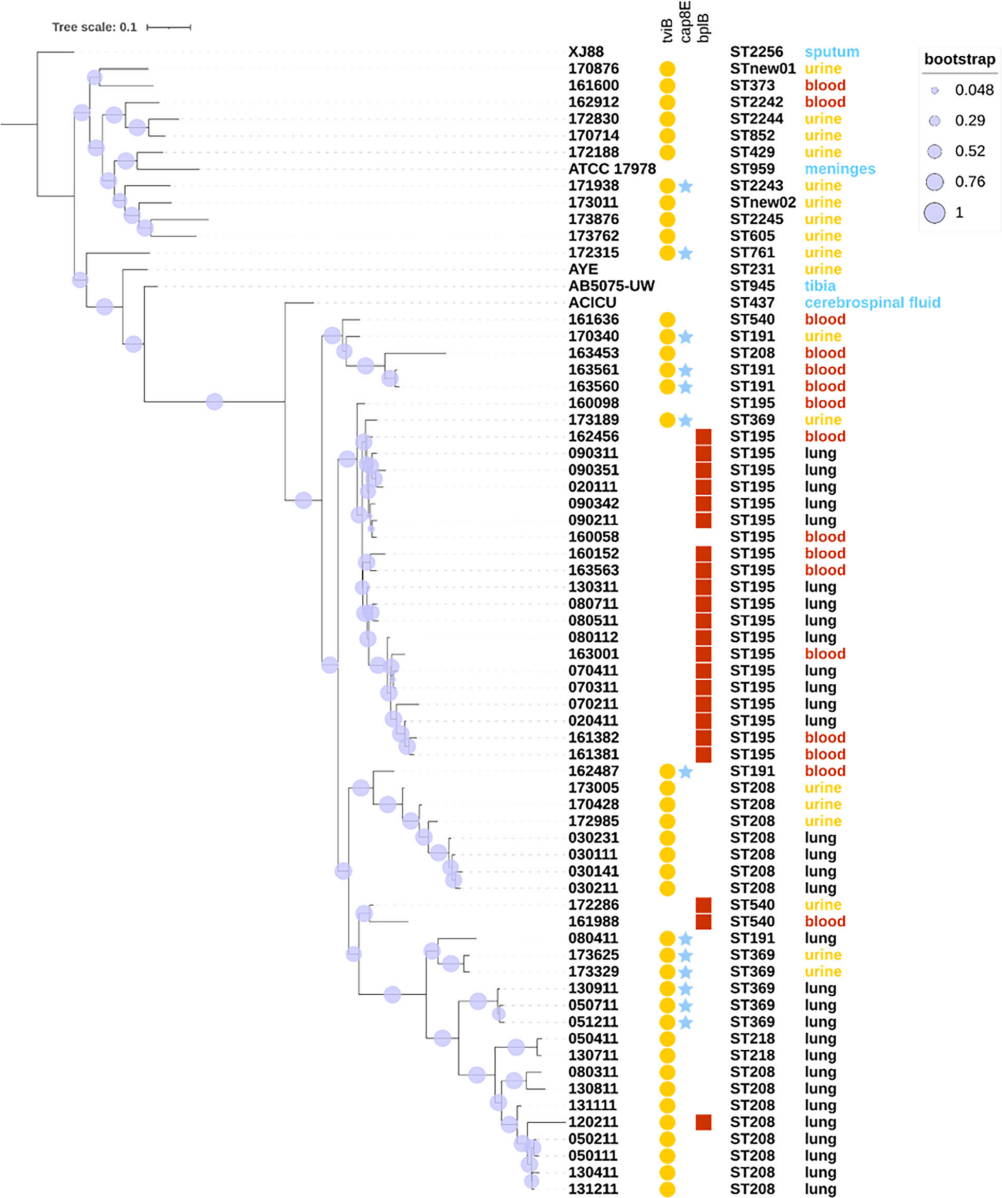
Cladogram of 68 *A. baumannii *and one*A. pitti* isolates based on non-core genes with infection sites, virulence genes and sequence types mapped to each isolate. The bootstrap values are represented by the purple circles on the branches. The branch length reflects the genetic distance of each strain

Multiple virulence factors were identified in *A. baumannii* such as *tviB* (polysaccharide biosynthesis), *cap8E* (capsular polysaccharide synthesis), *pilT, pilG* and *pilU* which are twitching motility proteins. Almost every isolate had at least one twitching motility protein which played a vital role in bacterial invasiveness and colonization. Twitching motility protein encoded gene *pilU* was widespread in all infection sites while *pilT* and *pilG* were both primarily identified in BSI and UTI (Figure S1). The gene *cap8E* involved in capsular polysaccharide synthesis had a higher positive rate in UTI (35.3 %) than in BSI (18.9 %) and HAP (12.9 %) although there was no significant difference (*p* = 0.18, chi-square test). The virulence factor *bplB* which encoded probable acetyltransferase was mostly identified in isolates belonging to ST195 (19/21, Fig. 3).

### MLST and eBURST analysis

**Fig. 4:**
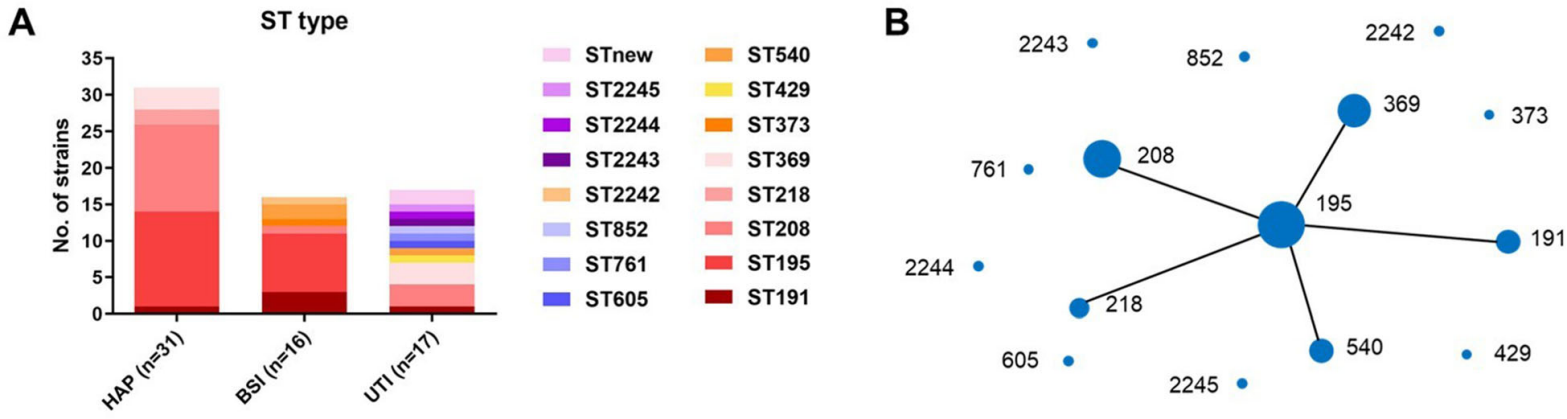
Sequence types of 64 *A. baumannii* isolates from three infection sites (**A**). eBURST analysis of 64 *A. baumannii* isolates (**B**). Each solid circle represents one sequence type and its size represents the quantity of isolates of this type. Each line between solid circles indicates one allele variation

Sixty-four *A. baumannii* isolates were divided into 11 STs according to the Oxford scheme. ST195 (21/64, 32.8 %) was the dominant sequence type followed by ST208 (16/64, 25.0 %), ST369 (6/64, 9.38 %), ST191 (5/64 7.81 %), ST540 (3/64, 4.69 %) and ST218 (2, 3.13 %). Each of the remaining sequence types (ST761, ST429, ST852, ST605 and ST373) had only one representative isolate. The six new STs were assigned sequence types from ST2242 ~ ST2245 and two strains were named STnew01 and STnew02 because of only 6/7 host genes. Figure 4 A displays the distribution of STs across each infection site. The HAP group was mainly composed of ST195 and ST208, while ST208 accounted for most of the BSI group. Isolates from the UTI group were more genetically diverse with 5 new STs. When using the Pasteur scheme, ST2 (53/64, 82.8 %) belonging to global clone II (GC2) accounted for the most of the sequence types.

The eBURST analysis depending on Oxford results showed that ST195 was the primary founder (Fig. 4B). ST195, ST208, ST369, ST191, ST540 and ST218 (53/64, 82.8 % in total) all belonged to clonal complex 208 (CC208, corresponding to GC2). The carbapenem resistance rates of CC208 and non-CC208 isolates were 96.2 and 9.1 %, respectively, suggesting that CC208 is a major epidemic clonal complex of carbapenem-resistant *A. baumannii*.

### Phylogenetic analysis

**Fig. 5:**
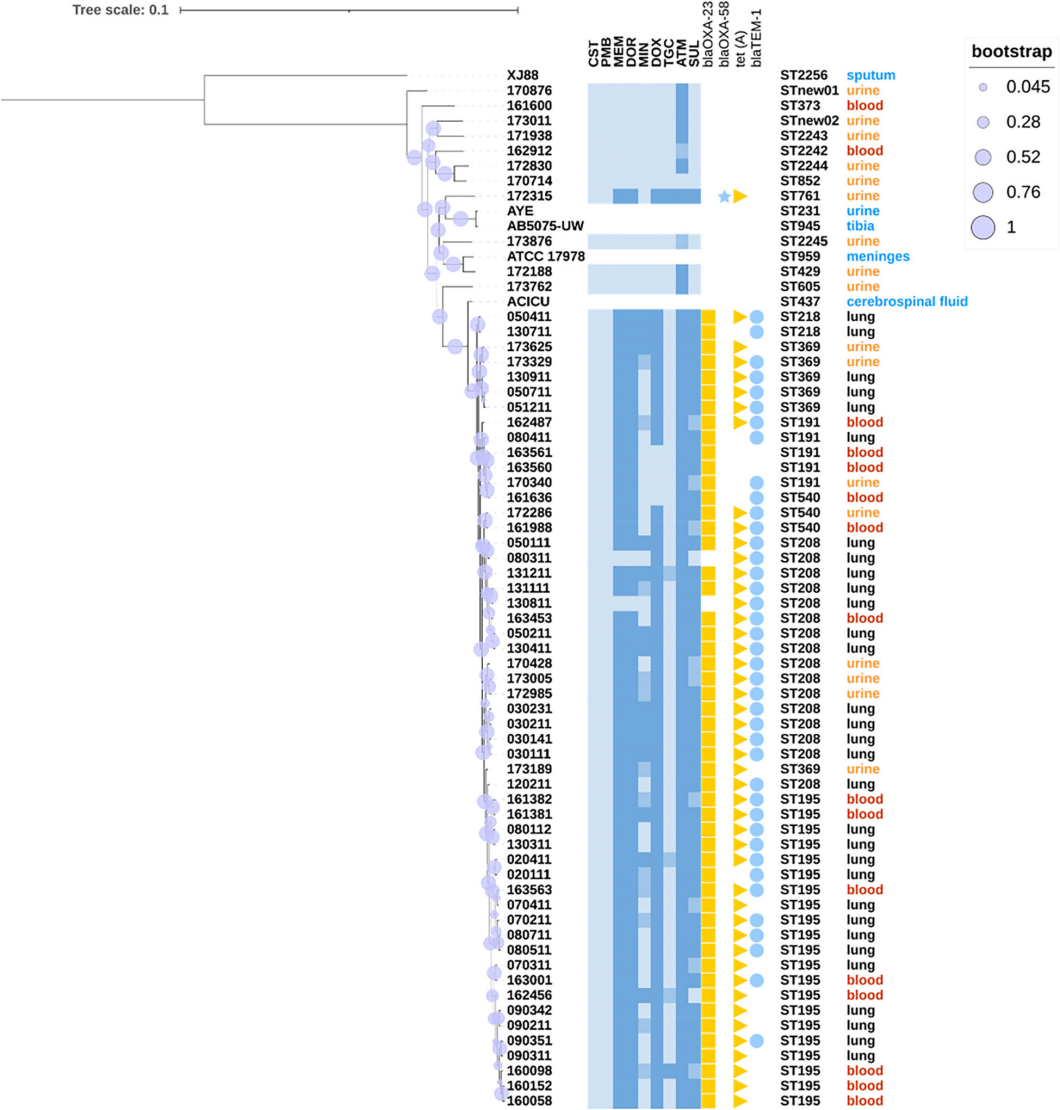
Core-gene based phylogenetic tree with sequence types, infection sites, resistance phenotype and genotype mapped to each of the 68 *A. baumannii* and one*A. pitti* isolates. The bootstrap values are represented by the purple circles on the branches. The branch length reflects the genetic distance of each strain. The branches from the top AB050411 to the bottom AB160058 belong to CC208. Antimicrobial susceptibility is distinguished by the color intensity as susceptible, intermediate and resistant (eg. the color dark blue represents resistance). For aztreonam and sulbactam without susceptibility breakpoints, MIC values were divided into ≤ 4, 8, ≥ 16 mg/L and ≤8, 16, ≥ 32 mg/L corresponding to the different color intensities, respectively. The susceptibility and resistance genes of ACICU, AYE, AB5075-UW, ATCC17978 and XJ88 were not provided. CST, colistin; PMB, polymyxin B; MEM, meropenem; DOR, doripenem; MIN, minocycline; DOX, doxycycline; TGC, tigecycline; ATM, aztreonam; SUL, sulbactam.

The core genome-based phylogenetic tree is presented in Fig. 5. It showed that clinical isolates from different infection sites were interspersed in the core-gene based phylogenetic tree rather than clustered together. All our clinical isolates were classified into two primary clades: CC208 and non-CC208. In the non-CC208 group, 9 of 11 were UTI isolates and 10 of 11 were carbapenem-susceptible. All of the CC208 isolates were carbapenem-resistant with *bla*_OXA−23_ gene, except HAP-isolates 080311 and 130,811 which demonstrated susceptibility to carbapenems without *bla*_OXA−23_. Intriguingly, one resistant isolate 172,315 from UTI carried carbapenemase gene *bla*_OXA−58_ instead of *bla*_OXA−23_. The positive rate of the tetracycline resistance gene *tet (A)* was 71.9 %, while that of *bla*_TEM−1_ responsible for monobactam resistance was 73.4 % (Fig. 5). *A. baumannii* isolates from public database were closer to our UTI isolates and *A. pitti* was far away from all the isolates.

In the cladogram, 69 *A. baumannii* isolates were separated into several clusters. Strictly speaking, these clusters were not well divided by infection sites or sequence types, however, strains with the same sequence type tend to cluster in near branches. Strikingly, virulence factor *cap8E* which encode capsular polysaccharide synthesis enzyme were positive in all isolates belonging to ST191 and ST369.

The SNP (Figure S2) and core-gene (Fig. 5) based phylogenetic tree display high consistency. Specifically, 53 isolates were clustered into CC-208 group with close genetic distance while the remaining 11 isolates belong to another cluster.

### AbGRI1 antibiotic resistance islands

Intact genomic islands were confirmed in 35 of 64 isolates (Table S2). The smallest island was 9.11 kb and the largest reached 37.8 kb. The GC contents in the islands ranged from 33.7 to 46.8 % (median 40.9 %), compared to 38.9 % of the whole genomes of 64 isolates.

**Fig. 6:**
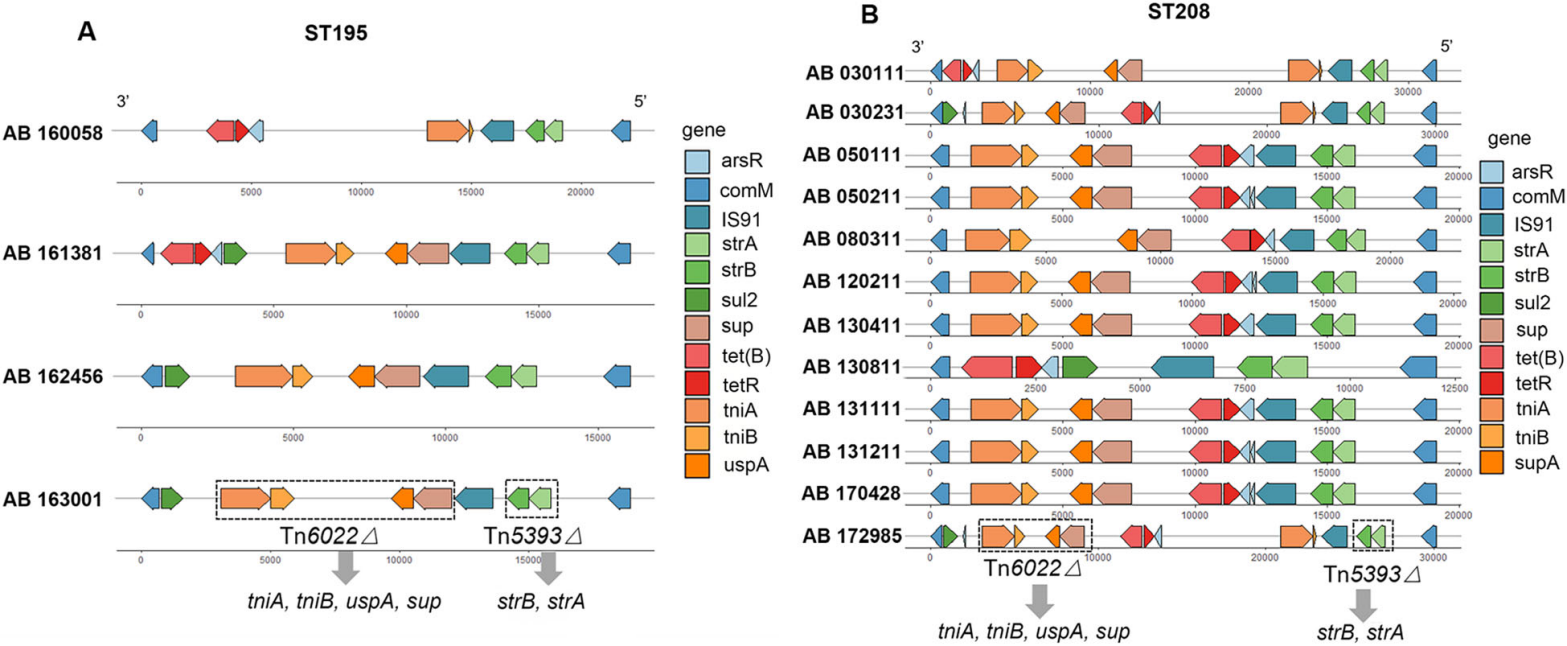
Genetic structures of ABGRI1 resistance islands in 16 strains belonging to ST195 (**A**) and ST208 (**B**). *arsR*, transcriptional regulator; *comM*, ATPase protein; IS91, insertion sequence 91; *strA, strB*, streptomycin phosphotransferase; *sul2*, sulfonamide resistance protein; *sup*, sulphate permease; *tet(B), tetR*, tetracycline resistance protein; *tniA*, transposase; *tniB*, NTP-binding protein; *uspA*, universal stress protein A

Among the 16 isolates from ST195 (*n* = 4) and ST208 (*n* = 12), 14 aside from 160058 and 130811 had identical Tn*6022△* structure carrying genes *tniA* (transposase), *tniB* (NTP-binding protein), *uspA* (universal stress protein) and *sup* (sulphate permease) (Fig. 6). The truncated Tn*5393△* structure with *strA* and *strB* (streptomycin phosphotransferase) was detected in all of the isolates near the 5’ end of the *comM* gene. The other genes, such as *tet(B)*, *tetR* and *sul2*, were located in the remaining regions conferring tetracycline and sulfonamide resistance. The genetic structures of the ABGRI1 resistance islands of the other 19 isolates are provided in Table S3.

The genetic context of the genomic islands was the same, indicating that the acquisition was ancestral to these 16 strains. The upstream genes encoded proteins included DNA modification methylase, diaminohydroxyphosphoribosylaminopyrimidine deaminase, ammonium transporter and ribonucleotide reductase transcriptional regulator NrdR. The downstream genes were involved in dihydrodipicolinate synthase family PA0223, ribulose-5-phosphate 4-epimerase and transcriptional regulator, GntR family domain.

### Relationship between single nucleotide polymorphisms (SNPs) and drug resistance

All identified SNPs were gene mutations with no insertion or deletion detected. Table S4 enumerates all the ns-SNPs that occurred in 64 *A. baumannii*. All of the ns-SNPs in the polymyxin resistance genes are listed in Table 1. The most frequently-occurring ns-SNPs had two amino acid substitutions in the histidine kinase gene *pmrB* which were V9I and I216T. The AA substitution also occurred in functional regions such as I216T in the HisK domain and Q344P in the HATPaseC domain. For the response regulator gene *pmrA*, mutations were discovered in the predicted receiver domain (A39T) and the unknown functional domain (S119T). Lipid A synthesis genes *lpxA* and *lpxC* had one (H131Y) and four (D159N, H149Y, D287N, M115I) amino acid substitutions, respectively, although no polymyxin resistance was detected with MICs ≤ 1 mg/L.

**Table 1:** Amino acid substitutions in polymyxin resistance genes

Amino acid changes									
*pmrA*^*d*^ (224AA)			*pmrB*^*d*^ (444AA)					*lpxA*	*lpxC*
Rec^a^ (AA 5 ~ 116)	AA 117 ~ 131		AA 1 ~ 215	HisK^b^ (AA 216 ~ 276)	AA 277 ~ 330	HATPaseC^c^ (AA 331 ~ 419)			
A39T	S119T		V9I; K105N; A146V	I216T	\	Q344P		H131Y	D159N; H149Y; D287N; M115I

^a^Rec, signal receiver domain; ^b^HisK, histidine kinase (dimerization/phosphoacceptor) domain; ^c^HATPaseC, histidine-kinase-like ATPase. ^d^Only domains or regions displaying mutations are shown. The amino acid (AA) positions corresponding to these domains are displayed in brackets

No nucleotide mutation was found in the carbapenemase gene *bla*_OXA−23_. Eleven of twelve non-CC208 isolates had the ns-SNPs in *bla*_OXA−66_ gene, although these 11 isolates exhibited susceptibility to carbapenems. It is noteworthy that three CC208 isolates belonging to ST369 which carries *bla*_OXA−23_ had the same non-synonymous mutation (F82L, I129L) in *bla*_OXA−66_ and displayed resistance to both meropenem and doripenem. They were identified as gene *bla*_OXA−83_ with single mutation (I129L) and *bla*_OXA−425_ with two mutations (F82L, I129L) in *bla*_OXA−66_ [9, 10]. The corresponding base change were 244T→C and 385 A→C. Nevertheless, in our cloning experiments, the MICs of *E.coli* top10 with mutation 244T→C remained constant or had an ≥ 2-fold decrease compared to wild-type *E.coli* top10. A 2-fold increase of the MICs was observed for *E.coli* top10 with mutation 385 A→C. For transformants with both mutations, the MICs remained the same or doubled (Table 2).

**Table 2:** The susceptibility of *E.coli* top10 and transformants to meropenem and doripenem

Isolate	MIC_MEM_ (mg/L)	MIC_DOR_ (mg/L)
*E.coli* top10_WT_	0.03	0.03
*E.coli* top10_244T→C_	≤ 0.015	0.03
*E.coli* top10_385A→C_	0.06	0.06
*E.coli* top10_244T→C,385 A→C_	0.03	0.06

*WT* wild type; *MEM* meropenem; *DOR* doripenem

In the MIC tests, two tigecycline resistant isolates (MIC = 8 mg/L) were identified. One ns-SNP of I100N in efflux pump gene *adeS* may explain the tigecycline-resistance of 160098 while for the remaining isolates (172315, 131211, 020411, 162456) that were non-susceptible to tigecycline, no AA substitution were identified in genes *adeR* and *adeS*.

## Discussion

*A. baumannii* is a common nosocomial pathogen, mostly causing hospital acquired pneumonia, bloodstream infection and urinary tract infection. However, the genomic characteristics, structural and functional relationship of *A. baumannii* from the three infection sites are still unknown. Investigating the epidemiological and genomic characteristics of these isolates provides reference for monitoring the antimicrobial resistance and virulence, thus reducing epidemiology of pathogenetic strains. In our study, we investigated the resistance and virulence characteristics of *A. baumannii* from HAP, BSI and UTI, analysing the probable resistance mechanisms and phylogenetic relationship of *A. baumannii*.

Previous studies have shown that *A. baumannii* belonging to ST195, ST208, ST191 and ST365 are the most widely spread in Chinese hospitals [4, 11]. The dominant types in our study were ST195, ST208, ST218 and ST191. Li [5] collected 52 clinical isolates of *A. baumannii* mainly from sputum as well as bloodstream. The proportion of multidrug and pan-drug resistant isolates in the CC208 group was not significantly different from that in the non-CC208 group. In our study, however, the carbapenem resistance rate of CC208 isolates was significantly higher than that of the non-CC208 group. Most studies have shown that *A. baumannii* CC208 outbreaks are highly correlated with the presence of the *bla*_OXA−23_ gene [12]. This was also confirmed in our study, in which 51 carbapenem-resistant isolates from the CC208 group (n = 53) were positive for the *bla*_OXA−23_ gene. CC208/GC2 was the largest clonal complex in *A. baumannii* which can often carry carbapenemase genes like *bla*_OXA−23−like_, *bla*_OXA−40−like_ and *bla*_OXA−58−like_. Compared to CC109/GC1 and CC187/GC3, CC208 was the only complex carrying three types of hydrolases; this could explain its wide spread to some extent [13]. It needs to be mentioned that MLST schemes are probably not the best ways to type *A. baumannii* due to its high genome variations. In order to evaluate the genetic relationship more accurately, we established phylogenetic relationship between strains of *A. baumannii* based on core genes, non-core genes, as well as SNPs. Strains are not well clustered according to infection sites, indicating that no specific core or accessary genes are responsible for different infections. In contrast, Liu [14] and Zhao et al. [15] claimed that unique genes may be closely associated with the ability of the bacteria to adapt to challenging niches. Our phylogenetic analysis also demonstrated that *A. baumannii* is a genomically variable pathogen that has the potential to cause a range of infectious diseases [16]. Considering the importance of phylogenetic relationship and sequence type, BacWGSTdb 2.0 (http://bacdb.cn/BacWGSTdb/) [17] provides a platform for rapid typing, phylogenetic relatedness linked to antibiotic resistance genes and clinical data, which is useful for further investigations.

Multiple factors contribute to the virulence of *A. baumannii* such as biofilm formation, motility, glycosylation and the micronutrient acquisition system [18]. In this study, motility proteins (*pilT, pilU, pilG*) and a polysaccharide biosynthesis protein (*tviB*) seemed to take a leading role in the fitness and colonization success of *A. baumannii.* Subashchandrabose and Wang et al. investigated about the genes necessary for persistence in the lung and for bacterial survival in bloodstream infection. Seven fitness genes were identified in these two studies, suggesting the presence of a core set of fitness genes irrespective of the site of infection [19, 20]. In the present study, the gene *pilU* was widespread in three infection sites while *pilT* and *pilG* genes tended to exist in BSI and UTI. It was noteworthy that virulence factors were likely associated with sequence types. The gene *cap8E* encoding capsular polysaccharide synthesis enzyme was identified in all isolates classified as ST191 and ST369. It has been recently reported that one of seven hypervirulent *A. baumannii* was identified as ST369 [21]. Further investigations are warranted to confirm the relationship between virulence genes and sequence types and monitor the key virulence factors associated with hypervirulence.

The multi-drug resistance of *A. baumannii* is a huge threat for clinical treatment and patient health. ABGRI1 resistance islands are a class of vital mobile genetic elements known to be involved in multiple antimicrobial resistance in *A. baumannii* GC2 [22]. *Tn6022* and *Tn6022△* were the most common transposons in AbGRI1. *Tn6022* consists of 7 known functional genes and 2 open reading frames and carries no resistance genes [22, 23]. In our study, the ABGRI1 resistance islands inserted in the *comM* gene shared similar backbones. *Tn6022△* consists of *tniA, tniB, uspA* and *sup* with *tniC, tniD* and *tniE* deleted compared to *Tn6022*. *Tn6022* has been shown to sometimes acquire the OXA-23 carbapenem resistance transposon *Tn2006* [24], though in our study *bla*_OXA−23_ may be located on mobile genetic elements like *Tn2009* or *Tn2006* which are the most common carriers and it is always plasmid-carried which enhances the spread of resistance [23]. Gene *bla*_OXA−23_ was not detected in ABGRI1 of our clinical isolates but it has been identified in Tn2006 in AbaR4 [24]. Genes associated with streptomycin, tetracycline and sulfonamide resistance were located on the genomic islands which indicates that ABGRI1 resistance island was not the only contributor to the MDR or carbapenem-resistant phenotype.

Oxacillinases are major causes of carbapenem resistance in *A. baumannii.* Within the CC208 group, the carbapenem-resistant isolates were all positive for *bla*_OXA−23_. For the three carbapenem-resistant isolates belonging to ST369 (050711, 051211, 130911), two non-synonymous mutations were both discovered in *bla*_OXA−66_ gene which referred to F82L and I129L. Previous studies have shown that enzyme OXA-66 can be converted to OXA-83, another subtype in the OXA-51 family, after the substitution of I129L. OXA-83 was first detected in two meropenem-resistant *A. baumannii* strains in the United Kingdom but the values of MIC were both 4 mg/L for imipenem [9]. In terms of tertiary protein structure, Ile-129 was close to the active site Ser-80 and the δ carbon of this isoleucine would cause a steric clash with the hydroxyethyl group of carbapenems [25] that was adverse to substrate binding. I129L relieved this clash, thus promoting carbapenem binding. This has been confirmed by molecular dynamics simulations [26]. When F82L and I129L substitutions both occurred in OXA-66, the subtype was specified as OXA-425. The OXA-425-positive and carbapenem resistant strain was first isolated in Beijing [10]. To the best of our knowledge, the influence of F82L and I129L on carbapenem resistance has not been confirmed by separate cloning experiments. Our study verified that both of these substitutions failed to cause carbapenem resistance. This suggests that the *bla*_OXA−23_ gene should be the most likely factor for carbapenem resistance in our *A. baumannii* isolates and mutational *bla*_OXA−66_ was not.

The resistance mechanism of *A. baumannii* to polymyxins is mainly regulated by two pathways. One is point mutations of the lipid A synthesis-related genes *lpxA, lpxC* and *lpxD*, which inhibits the synthesis of lipid A [27]. Another mechanism is regulated by the two-component system of *pmrAB*. Studies have shown that point mutations in *pmrB* and the subsequent upregulation of *pmrAB* are critical for polymyxin resistance [28]. Resistance-related point mutations are mainly located in the histidine kinase domain (HisK, AA 216 ~ 276) and the ATP binding domain (HATPaseC, AA 331 ~ 419) of *pmrB* [29]. Resistance caused by point mutations of *pmrA* has also been reported [30], especially in the signal receiver domain (Rec). In this study, several non-synonymous mutations occurred in the *pmrAB* functional domain demonstrating that not all non-synonymous mutations in the *pmrAB* functional region cause resistance. Amino acid changes in the PmrAB two-component system have been suggested not essential for *A. baumannii* colistin resistance [31]. Meanwhile, no resistance occurred in the mutants with ns-SNPs in the *lpxA* and *lpxC* genes.

Over-expression of AdeABC efflux pump stimulated continuously by the mutated AdeRS two component system has been found to result in tigecycline resistance. The AA substitution in gene *adeS* (I100N) is probably responsible for higher MIC of isolate 160098. For remaining tigecycline non-susceptible isolates, mutations in regulatory genes of resistance-nodulation cell division efflux pumps such as *adeN, adeJ* and ISaba1 insertion into genes *adeN* and *adeRS* may explain the tigecycline resistance [32–34].

## Conclusions

In summary, our study sheds new light on the epidemiological characteristics and phylogenetic relationship of clinical *A. baumannii* across China and uncovers the possible molecular mechanisms of multi-drug resistance and virulence. The *bla*_OXA−23_ gene is probably responsible for high proportion of carbapenem resistance. CC208 was the predominant clone in *bla*_OXA−23_-carrying *A. baumannii*. Several key virulence factors such as *cap8E* also deserves attention. Multiple phylogenetic analysis indicates that *A. baumannii* is a genomically variable pathogen that has the potential to cause a range of infectious diseases. All of the evidence indicates that the resistance and virulence should be monitored to reduce the resistance transmission and mortality caused by probably hypervirulent *A. baumannii.*

## Methods

### Bacterial isolates and antimicrobial susceptibility testing

In this study, clinical *A. baumannii* isolates from BSI (*n* = 17) and UTI (*n* = 16) were collected from Huashan Hospital in Shanghai between 2016 and 2017. Isolates from HAP (*n* = 31) were from a domestic thirteen-centre clinical study on colistin methanesulfonate (registration number: NCT01940731) including Hunan People’s Hospital, Shanghai 10th People’s Hospital, The Second Hospital of Jilin University, West China Hospital of Sichuan University, etc. across China. Colistin sulfate (lot number SLBD8306V; Sigma-Aldrich, St Louis, MO), polymyxin B (lot number R046V0; USP), minocycline, doxycycline, sulbactam, meropenem, aztreonam (lot numbers: 130514–200401, 130485–201703, 130430–201408, 130506–201403 and 130507–201303, respectively; National Institutes for Food and Drug Control, Beijing, China), tigecycline (lot number: 10-MWC-62-1; USP) and doripenem (lot number 0379; Shionogi & Co Ltd) were used in this study. The minimum inhibitory concentrations (MICs) of 64 isolates were determined using the microbroth dilution method for three replicates in one batch with *Escherichia coli* ATCC25922 as the quality control. A total of nine antimicrobials were included and the results were interpreted referring to the CLSI where possible (CLSI M100 2020). CLSI does not currently provide breakpoints for tigecycline, sulbactam and aztreonam. FDA-recommended criteria for *Enterobacteriaceae* were used for tigecycline susceptibility (≤ 2, 4, ≥ 8 mg/L) [35].

### DNA extraction and whole genome sequencing

The genome DNA of 64 isolates were extracted according to the Takara DNA Extraction Kit protocol. The whole genome was sequenced using the Illumina Hiseq X10 platform, with the 2*150 bp paired-end sequencing strategy [36]. The raw read data were assembled de novo using Velvet software [37].

### Functional annotation of resistance and virulence genes

For functional classification of the predicted core genes, BLASTP was used to align amino acids of predicted genes against the Clusters of Orthologous Groups (COG) database with an expected threshold of 1E^− 3^ using the Conserved Domains Database (CDD) [38]. We also performed sequence alignment of the amino acid sequences to the NCBI non-redundant (NR) database (E-value ≤ 1E^− 3^).

In order to identify antibiotic resistance genes, the protein-coding sequences were aligned against the Comprehensive Antibiotic Resistance Database (CARD, https://card.mcmaster.ca/) [39]. The similarity and length consistency were required to be over 80 %. To search possible virulence factors, the Virulence Factors Database (VFDB, http://www.mgc.ac.cn/VFs/) [40] was aligned to the ORF protein sequences, using the same thresholds as alignment of resistance genes. Single nucleotide polymorphisms in genes were investigated including *lpxA, lpxC, pmrA, pmrB* (polymyxin resistance), *bla*_OXA−23_, *bla*_OXA−66_ (carbapenem resistance), *carO, oprD* (efflux pump), *bla*_Tem−1_ (monobactam, penem), *tet (A), adeR, adeS* (tetracycline, glycylcycline). Only non-synonymous SNPs (ns-SNPs) were included in the analysis.

### Multilocus sequence typing (MLST) and eBURST analysis

To determine the sequence types, multi-locus sequence typing (MLST) was performed according to both the Oxford and Pasteur schemes. Sequences were compared to the PubMLST database for *A. baumannii* (http://pubmlst.org/abaumannii/) and then assigned to the appropriate sequence types. The genome submission to PubMLST was completed for all the 64 isolates (submission ID: BIGSdb_20201227120226_136766_49027). The following four isolates were assigned new STs: 162912 (ST2242), 171938 (ST2243), 172830 (ST2244) and 173876 (ST2245). Another two isolates 170876 and 173011 had only 6/7 host genes, they can not be assigned new STs in PubMLST. Thus, they were named STnew01 and STnew02.

The eBURST analysis of the isolates was performed to determine their homology [41]. Isolates sharing 6/7 alleles were considered to be a single clonal complex (CC) group.

### Phylogenetic analysis

Phylogenetic trees were constructed using three strategies: gene-by-gene genomic analysis (cgMLST), non-core gene-based strategy (cladogram) and a reference genome-based single nucleotide polymorphism (SNP) strategy. Four *A. baumannii* isolates (ATCC17978, AYE, ACICU, AB5075-UW) and one *A. pitti* isolate (XJ88) from public database were included in the analysis. The detailed information about these isolates is provided in Table S5. Raw sequence data from all clinical isolates were independently mapped to the reference isolate *A. baumannii* AC30 genome sequence (accession number: CP007577.1) using bowtie2 (http://bowtie-bio.sourceforge.net/bowtie2/index.shtml). *A. baumannii* AC30 belongs to ST 195 which is the most epidemic sequence type in our study. GATK (https://gatk.broadinstitute.org/hc/en-us) was utilized to identify mutations such as SNPs and INDEL. High-quality single nucleotide polymorphisms (SNPs) were selected with a mapping coverage of > 10 and a frequency of > 70 %. The multiple sequence alignment was performed using MAFFT and phylogenetic trees were constructed with FastTree following the maximum likelihood method. The cladogram was constructed according to the presence /absence of non-core genes based on the pangenome analysis using the software ROARY. The iTOL v5 (https://itol.embl.de/) was utilized for integrating the phylogenetic tree with sequence types, infection sites, resistance phenotype and genotype and virulence factors.

### ABGRI1 resistance island analysis

The ABGRI1 inserted in the *comM* gene was extracted and analysed. An intact ABGRI1 could be split on different contigs in draft genomes; thus only intact ABGRI1 islands were chosen for further comparative analysis. The annotations were performed with RAST (https://rast.nmpdr.org/) and genomic island structure was constructed using R 3.5.0 for isolates belonging to ST195 and ST208.

### Cloning experiment

Cloning experiments were conducted to determine the effects of non-synonymous mutations in *bla*_OXA− 66_ gene on carbapenem susceptibility. In order to clone *bla*_OXA− 66_ into pHSG398 in the proper orientation, restriction sites BamHI and PstI in the multiple cloning sites downstream of the pLac promoter of pHSG398 were chosen to insert the 5’-end and 3’-end of *bla*_OXA− 66_, respectively. Primers were designed according to the principles listed in the In-Fusion® HD Cloning Kit User Manual (Takara Bio USA, Inc): adding 15 to 20 bp homologous sequences of linearized vector to the 5’-ends of both forward and reverse primers to make the ends of amplified inserts and linearized vectors identical to each other (Table 3). Thus, primers BamHI-*bla*_OXA− 66_-FW and PstI-*bla*_OXA− 66_-RW were used to amplify the *bla*_OXA− 66_ gene. Purified PCR product of *bla*_OXA− 66_ gene and the linearized pHSG398 vector, which were digested by BamHI and PstI enzymes, were mixed at an appropriate ratio and incubated with 5× In-Fusion HD Enzyme Premix at 50℃ for 15 min. Then the recombination product was transformed into competent cell *E.coli* Top10 by heat-shock and screened on plates containing 50 mg/L chloramphenicol. The clones with *bla*_OXA− 66_ were further confirmed by PCR and sequencing.

**Table 3:** Primers used in the cloning experiment

Primer	5’-3’ sequence	Source
BamHI-*bla*_OXA−66_-FW	CGAATTCGAGCTCGGTACCCGGGGATCCATGAACATTAAAGCACTCTTACTT	This study
PstI-*bla*_OXA−66_-RW	CCAGTGCCAAGCTTGCATGCCTGCAGCTATAAAATACCTAATTGTTCTAAG	This study
T244C-RW	CAAAGCATTAAGCATTTTGA**G**GGTCGAAGCAGGTACATACTC	This study
T244C-FIW	TCAAAATGCTTAATGCTTTG	This study
A385C-RW	CTAAATCTTGATAAACTGGAA**G**AGCGGAAGCTTTCATGGCATC	This study
A385C-FIW	TTCCAGTTTATCAAGATTTAGCTCGTCG	This study

BamHI and PstI restriction sites were underlined

For the *bla*_OXA−66_ alleles with T244C mutation, similar cloning experiment was performed except that the insert gene was amplified with two pairs of PCR primers: BamHI-*bla*_OXA−66_-FW and T244C-RW for the first 264 bp of *bla*_OXA−66_ gene with T244C mutation, and T244C-FIW and PstI-*bla*_OXA−66_-RW for the last 581 bp of *bla*_OXA−66_ gene. Both the two purified PCR fragments were mixed with linearized pHSG398 vector at an appropriate ratio together with 5×In-Fusion HD Enzyme Premix for recombination reaction.

As for the *bla*_OXA−66_ allele with T244C and A385C mutations, three pairs of primers were used for amplification: BamHI-*bla*_OXA−66_-FW and T244C-RW, T244C-FIW and A385C-RW, and A385C-FIW and PstI-*bla*_OXA−66_-RW.

The bases in the box were designed for the mutation of T244C and A385C.

## Accession numbers

Accession numbers of 64 *A. baumannii* are provided in Table S4.

## Supplementary Information

**Additional file 1:**

**Additional file 2:**

**Additional file 3:**

**Additional file 4:**

**Additional file 5:**

## Data Availability

The datasets analysed in this study are all available in NCBI (BioProject number PRJNA633416, https://www.ncbi.nlm.nih.gov/bioproject/PRJNA633416).

## Abbreviations

HAP: Hospital acquired pneumonia
BSI: Bloodstream infection
UTI: Urinary tract infection
CC: Clonal complex
XDRAB: Extensively drug-resistant *A. baumannii*
MLST: Multilocus sequence typing
SNP: Single nucleotide polymorphisms
MIC: Minimum inhibitory concentration
AA: Amino acid
