# Correction to: epidemiological and genomic characteristics of *A. baumannii* from different infection sites using comparative genomics

**DOI:** 10.1186/s12864-021-07942-2

**Published:** 2021-09-08

**Authors:** Xingchen Bian, Xiaofen Liu, Xuefei Zhang, Xin Li, Jing Zhang, Huajun Zheng, Sichao Song, Xiang Li, Meiqing Feng

**Affiliations:** 1 School of Pharmacy & Minhang Hospital, Fudan University, 826 Zhang Heng Rd, Shanghai, 201203 China; 2 Institute of Antibiotics, Huashan Hospital, Fudan University, Shanghai, 200040 China; 3 Key Laboratory of Clinical Pharmacology of Antibiotics, Shanghai, 200040 China; 4 Huashan Hospital, National Health Commission & National Clinical Research Center for Aging and Medicine, Fudan University, Shanghai, 200040 China; 5 Phase I Unit, Huashan Hospital, Fudan University, Shanghai, 200040 China; 6 Chinese National Human Genome Center, Shanghai, 201203 China

**Correction to:** ***BMC Genomics*** **22, 530 (2021).**

10.1186/s12864-021-07842-5

Following publication of the original article [1], it was reported that there was an error in Fig. 4. The image used was a duplicate of that for Fig. 5. The correct Fig. 4 is provided in this Correction article and the original article has been updated.

**Fig. 4:**
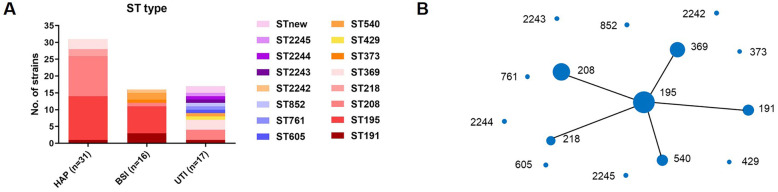
Sequence types of 64 *A. baumannii* isolates from three infection sites (**A**). eBURST analysis of 64 *A. baumannii* isolates (**B**). Each solid circle represents one sequence type and its size represents the quantity of isolates of this type. Each line between solid circles indicates one allele variation
