# Supplementary material for: Epidemiological and genomic characteristics of Acinetobacter baumannii from different infection sites using comparative genomics
Source: BMC Genomics. 2021 Jul 12;22:530. doi: 10.1186/s12864-021-07842-5 (PMC8272988; doi:10.1186/s12864-021-07842-5)
Supplement: Supplementary file 1 — Additional file 1: [file 12864_2021_7842_MOESM1_ESM.docx]

**Epidemiological and** **genomic characteristics of *Acinetobacter baumannii* from different infection sites using comparative genomics**

Xingchen Bian^1,2,3,4^, Xiaofen Liu^2,3,4^, Xuefei Zhang^2^, Xin Li^2,3,4^, Jing Zhang^2,3,4,5^, Huajun Zheng^6^, Sichao Song^6^, Xiang Li^1*^, Meiqing Feng^1*^

^1^ School of Pharmacy & Minhang Hospital, Fudan University, Shanghai, 201203, China;

^2^ Institute of Antibiotics, Huashan Hospital, Fudan University, Shanghai, 200040, China;

^3^ Key Laboratory of Clinical Pharmacology of Antibiotics, Shanghai, 200040, China;

^4^ National Health Commission & National Clinical Research Center for Aging and Medicine, Huashan Hospital, Fudan University, Shanghai, 200040, China;

^5^ Phase I Unit, Huashan Hospital, Fudan University, Shanghai, 200040, China;

^6^ Chinese National Human Genome Center, Shanghai, 201203, China;

^*^Corresponding author: Meiqing Feng, 826 Zhang Heng Rd, Shanghai, 201203, China; Email: [fmq@fudan.edu.cn](mailto:fmq@fudan.edu.cn) ; Xiang Li, 170 Xinsong Rd, 201100, China; Email: [xiangli_mh@fudan.edu.cn](mailto:xiangli_mh@fudan.edu.cn)

**Running title**: genomic characteristics of *Acinetobacter baumannii*

**Key words**: *Acinetobacter baumannii*, whole genome sequencing, epidemiological characteristics, multi-drug resistance, comparative genomics

**Figure S1.** Virulence factors in 64 *A. baumannii* isolates. *htp8*, Hsp60, 60K heat shock protein HtpB; *orf18*, Unknown; *orf48, orf66,* hypothetical protein; *pilT, pilU, pilG,* twitching motility protein; *bplB*, probable acetyltransferase; *tetA(B),* tetracycline resistance protein TetA(B); *tetR,* tet repressor; *tviB*, Vi polysaccharide biosynthesis protein, UDP-glucose/GDP-mannose dehydrogenase; *aadA1*, streptomycin adenyltransferase AadA1; *intl1*, Tn21 integrase IntI1; *tnpR*, resolvase TnpR; *cap8E*, capsular polysaccharide synthesis enzyme Cap8E; *VC0817*, transposase, putative

**
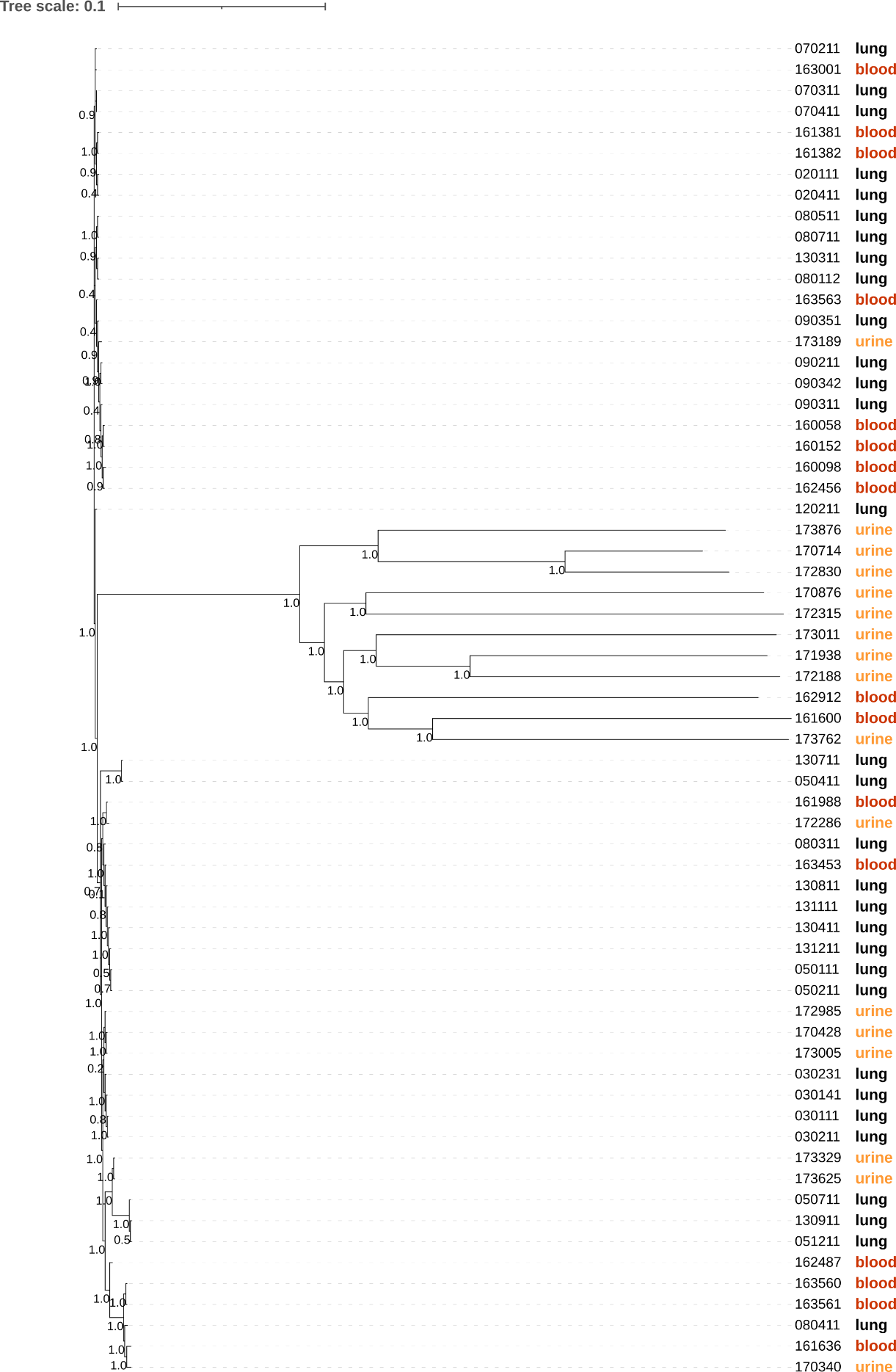
**

**Figure S2.** SNP-based phylogenetic tree of 64 *A. baumannii* isolates with infection sites.

**Table S2.** The full length and GC contents of intact AbGRI1 islands

| Infection type | Strain | Total base (bp) | GC (%) | Infection type | Strain | Total base (bp) | GC (%) |
| --- | --- | --- | --- | --- | --- | --- | --- |
| HAP | 030111 | 30193 | 38.3 | HAP | 131211 | 17595 | 40.9 |
|  | 030231 | 28537 | 39.4 | BSI | 160058 | 20701 | 39.0 |
|  | 050111 | 17595 | 40.9 |  | 161381 | 16930 | 43.1 |
|  | 050211 | 17595 | 40.9 |  | 161600 | 11963 | 36.1 |
|  | 050411 | 20648 | 40.3 |  | 161636 | 16441 | 36.0 |
|  | 050711 | 17595 | 40.9 |  | 162456 | 14527 | 43.3 |
|  | 051211 | 17595 | 40.9 |  | 162487 | 12060 | 36.0 |
|  | 080311 | 20445 | 40.3 |  | 163001 | 17389 | 42.3 |
|  | 080411 | **9113** | 35.7 |  | 163561 | 18527 | 36.2 |
|  | 090311 | 18496 | 41.1 | UTI | 170340 | 21930 | 36.0 |
|  | 090342 | 22007 | 41.3 |  | 170428 | 17595 | 40.9 |
|  | 090351 | 18614 | 41.1 |  | 170876 | **37800** | 37.0 |
|  | 120211 | 17790 | 40.8 |  | 172286 | 20445 | 40.3 |
|  | 130411 | 17595 | 40.9 |  | 172985 | 28616 | 39.4 |
|  | 130711 | 31488 | 39.0 |  | 173329 | 17595 | 40.9 |
|  | 130811 | 10544 | **46.8** |  | 173625 | 17595 | 40.9 |
|  | 130911 | 17595 | 40.9 |  | 173762 | 19781 | **33.7** |
|  | 131111 | 17595 | 40.9 |  | **median** | **17595** | **40.9** |

**Table S5.** The information of *A. baumannii* and *A. pitti* isolates from public database

| Strain | Country | Year | Source | MLST (Oxford) | MLST  (Pasteur) |
| --- | --- | --- | --- | --- | --- |
| ATCC17978 | France | 1951 | Meninges | 959 | 437 |
| AYE | France | 2001 | Urine | 231 | 1 |
| ACICU | Italy | 2005 | CSF | 437 | 2 |
| AB5075-UW | USA | 2008 | Tibia/  Osteomyelitis | 945 | 1 |
| XJ88(*A. pitti*) | China | 2016 | Sputum | 2256 | 485 |
