# Supplementary material for: Epidemiological and genomic characteristics of Acinetobacter baumannii from different infection sites using comparative genomics
Source: BMC Genomics. 2021 Jul 12;22:530. doi: 10.1186/s12864-021-07842-5 (PMC8272988; doi:10.1186/s12864-021-07842-5)
Supplement: Supplementary file 3 — Additional file 3: [file 12864_2021_7842_MOESM3_ESM.docx]

**Table S2.** The full length and GC contents of intact AbGRI1 islands

| Infection type | Strain | Total base (bp) | GC (%) | Infection type | Strain | Total base (bp) | GC (%) |
| --- | --- | --- | --- | --- | --- | --- | --- |
| HAP | 030111 | 30193 | 38.3 | HAP | 131211 | 17595 | 40.9 |
|  | 030231 | 28537 | 39.4 | BSI | 160058 | 20701 | 39.0 |
|  | 050111 | 17595 | 40.9 |  | 161381 | 16930 | 43.1 |
|  | 050211 | 17595 | 40.9 |  | 161600 | 11963 | 36.1 |
|  | 050411 | 20648 | 40.3 |  | 161636 | 16441 | 36.0 |
|  | 050711 | 17595 | 40.9 |  | 162456 | 14527 | 43.3 |
|  | 051211 | 17595 | 40.9 |  | 162487 | 12060 | 36.0 |
|  | 080311 | 20445 | 40.3 |  | 163001 | 17389 | 42.3 |
|  | 080411 | **9113** | 35.7 |  | 163561 | 18527 | 36.2 |
|  | 090311 | 18496 | 41.1 | UTI | 170340 | 21930 | 36.0 |
|  | 090342 | 22007 | 41.3 |  | 170428 | 17595 | 40.9 |
|  | 090351 | 18614 | 41.1 |  | 170876 | **37800** | 37.0 |
|  | 120211 | 17790 | 40.8 |  | 172286 | 20445 | 40.3 |
|  | 130411 | 17595 | 40.9 |  | 172985 | 28616 | 39.4 |
|  | 130711 | 31488 | 39.0 |  | 173329 | 17595 | 40.9 |
|  | 130811 | 10544 | **46.8** |  | 173625 | 17595 | 40.9 |
|  | 130911 | 17595 | 40.9 |  | 173762 | 19781 | **33.7** |
|  | 131111 | 17595 | 40.9 |  | **median** | **17595** | **40.9** |
